# Multiplex electrochemical sensing platforms for the detection of breast cancer biomarkers

**DOI:** 10.3389/fmedt.2024.1360510

**Published:** 2024-02-15

**Authors:** Connor O’Brien, Chun Keat Khor, Sina Ardalan, Anna Ignaszak

**Affiliations:** ^1^ Faculty of Medicine, Memorial University of Newfoundland, St. John’s, NL, Canada; ^2^ Department of Chemistry, University of New Brunswick, Fredericton, NB, Canada

**Keywords:** breast cancer, serum biomarker, multiplex, electrochemical biosensor, point-of-care POC

## Abstract

Herein, advancements in electroanalytical devices for the simultaneous detection of diverse breast cancer (BC) markers are demonstrated. This article identifies several important areas of exploration for electrochemical diagnostics and highlights important factors that are pivotal for the successful deployment of novel bioanalytical devices. We have highlighted that the limits of detection (LOD) reported for the multiplex electrochemical biosensor can surpass the sensitivity displayed by current clinical standards such as ELISA, FISH, and PCR. HER-2; a breast cancer marker characterised by increased metastatic potential, more aggressive development, and poor clinical outcomes; can be sensed with a LOD of 0.5 ng/ml using electrochemical multiplex platforms, which falls within the range of that measured by ELISA (from picogram/ml to nanogram/ml). Electrochemical multiplex biosensors are reported with detection limits of 0.53 ng/ml and 0.21 U/ml for MUC-1 and CA 15-3, respectively, or 5.8 × 10^−3^ U/ml for CA 15-3 alone. The sensitivity of electrochemical assays is improved when compared to conventional analysis of MUC-1 protein which is detected at 11–12 ng/ml, and ≤30 U/ml for CA 15-3 in the current clinical blood tests. The LOD for micro-ribonucleic acid (miRNA) biomarkers analyzed by electrochemical multiplex assays were all notedly superior at 9.79 × 10^−16^ M, 3.58 × 10^−15^ M, and 2.54 × 10^−16^ M for miRNA-155, miRNA-21, and miRNA-16, respectively. The dogma in miRNA testing is the qRT-PCR method, which reports ranges in the ng/ml level for the same miRNAs. Breast cancer exosomes, which are being explored as a new frontier of biosensing, have been detected electrochemically with an LOD of 10^3^–10^8^ particles/mL and can exceed detection limits seen by the tracking and analysis of nanoparticles (∼ 10^7^ particles/ml), flow cytometry, Western blotting and ELISA, etc. A range of concentration at 78–5,000 pg/ml for RANKL and 16–1,000 pg/ml for TNF is reported for ELISA assay while LOD values of 2.6 and 3.0 pg/ml for RANKL and TNF, respectively, are demonstrated by the electrochemical dual immunoassay platform. Finally, EGFR and VEGF markers can be quantified at much lower concentrations (0.01 and 0.005 pg/ml for EGFR and VEGF, respectively) as compared to their ELISA assays (EGRF at 0.31–20 ng/ml and VEGF at 31.3–2,000 pg/ml). In this study we hope to answer several questions: (1) Are the limits of detection (LODs) reported for multiplex electrochemical biosensors of clinical relevance and how do they compare to well-established methods like ELISA, FISH, or PCR? (2) Can a single sensor electrode be used for the detection of multiple markers from one blood drop? (3) What mechanism of electrochemical biosensing is the most promising, and what technological advancements are needed to utilize these devices for multiplex POC detection? (4) Can nanotechnology advance the sensitive and selective diagnostics of multiple BC biomarkers? (5) Are there preferred receptors (antibody, nucleic acid or their combinations) and preferred biosensor designs (complementary methods, sandwich-type protocols, antibody/aptamer concept, label-free protocol)? (6) Why are we still without FDA-approved electrochemical multiplex devices for BC screening?

## 1 Introduction

Accounting for a quarter of all cancers in women, breast cancer (BC), a notoriously heterogeneous disease, is the most commonly present cancer in women globally (1). Currently, BC screening occurs using the gold standards of mammography and routine breast examination; each of which presents challenges in sensitivity and specificity. Unfortunately, overdiagnosis/false positives are a risk associated with mammography that can result in increased patient anxiety and, at times, unnecessary biopsy (2). Additionally, mammography presents several confounding variables such as physician interpretation, poor positioning, and tumour subtypes that can result in as many as 30% of breast cancers going undetected (3). Routine breast examinations on the other hand are limited to the ability of the physician and the patient to both identify and investigate changes to the breast over time. Those with dense breast tissue, approximately 43% of women, are at an even greater risk of diagnostic mishap and thus subsequent distress and harm caused by untimely intervention (4, 5). As such, the search for different screening methods with better specificity and lower false-positive rates is of great interest.

There are various risk factors associated with the incidence of BC including family history, age, geographic location, environmental pollutants, socioeconomic status, lifestyle (consumption of alcohol, diet, lack of physical activity) and increased breast density (6). For women of all ages and at average risk, more frequent screening (mammography and clinical breast examination, CBE) could reduce mortality by 20% (7). In fact, when caught in the early stages of disease BC presents with good clinical outcomes and is curable in 70%–80% of all cases (8). Although the majority of patients diagnosed with new onset BC are postmenopausal (9), young women (defined as <40 years old) and premenopausal women generally present with more aggressive disease and poorer outcomes than the older population. Although current screening standards have been integral to the successful identification and treatment of BCs, there are ways we may yet improve the efficiency of screening programs and reduce the burden of screening for those at an increased risk of BC. Current screening methods pose risks of both false positive and false negative results—especially in those at-risk who present with dense breast tissue (10). These complications in detection may be supplemented using circulating biomarker detection panels which tend to be specific and accurate while allowing for a wide range array of serological differentiation.

It is well established in the literature that early detection is essential to improve outcomes and reduce mortality. Ensuring timely, affordable, and effective cancerous biomarker monitoring is the key to the successful implementation of cancer screening programs (11). The concept of electrochemical biosensing can be utilized to accomplish each of these as testing is generally rapid (within minutes), sensitive (a single cell detection or atto-/femto-molar concentrations of biomarker), and portable (most electrochemical readers are battery-operated and miniaturized to a table or even the pocket-size). By measuring the change in electrochemical parameters such as resistivity (impedance spectroscopy) or current (amperometry and voltammetry) (12–15), redox reactions at the electrode or membrane surfaces can be detected and immediately interpreted for diagnostic results (9, 16). The core element of the electrochemical assay is its biorecognition element (receptor), which can be aptamers, antibodies, enzymes, or proteins, which are immobilized onto an electrical transducer (electrode). The receptor forms a three-dimensional structure that functions as a ligand which binds to its associated analyte (biomarker) with high specificity. Upon binding, the conformation-changing property of the receptor structure can be utilized in biosensors through a redox-active molecule (redox probe). This probe either covalently attaches to the receptor on the electrode surface or can be added to the measurement solution. Once it is presented with a biomarker-rich medium, binding occurs and the current passing through the transducer (electrode) changes proportionately in real-time (13). Furthermore, the binding event is transduced into an electronic signal that quantifies the amount of analyte present in a sample and the electrochemical output can be immediately interpreted for diagnostic results.

**Figure 1:**
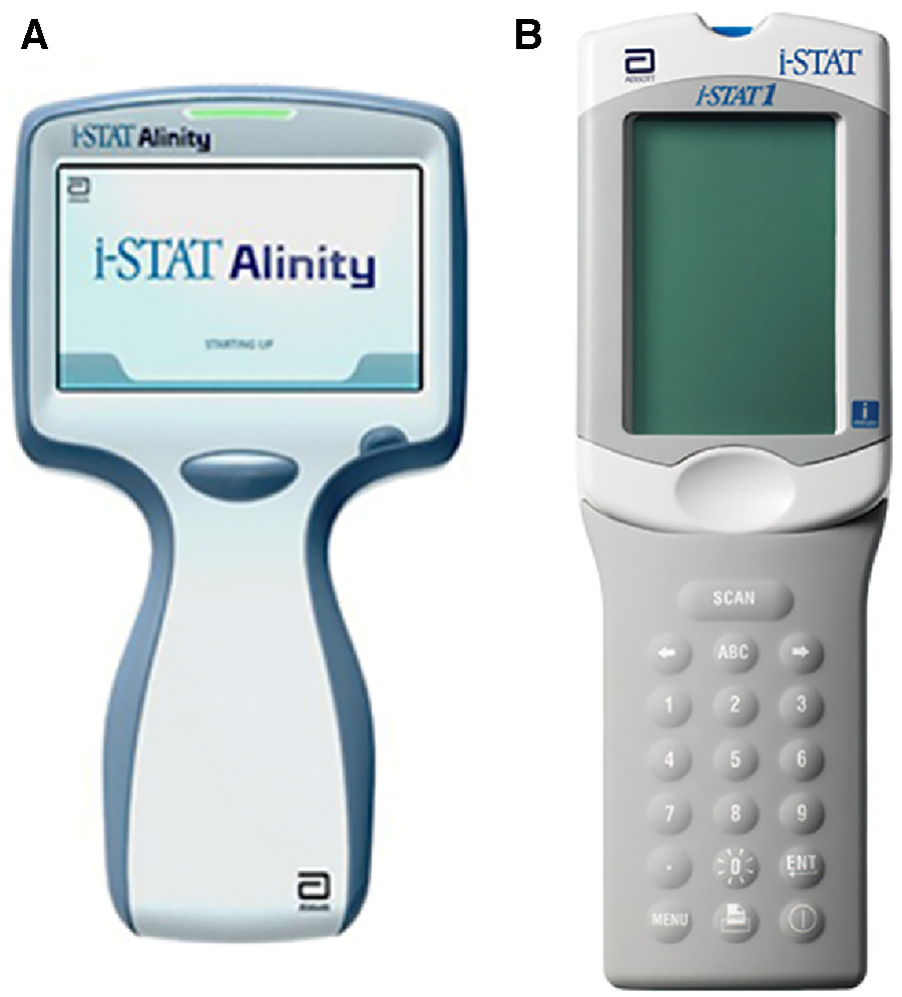
(**A**) Abbotts’ hand-held portable iSTAT alinity multiplex electrochemical tester for blood TBI (traumatic brain injury + blood gases, chemistries, electrolytes and haematology) biomarkers and (**B**) iSTAT 1 electrochemical multiplex blood tester. Copyright permissions granted by Abbott.

Although effective, there is a distinct disadvantage in using a simple electrochemical sensor as it is designed to interact with, and therefore detect, only one biomolecule at a time. This is where the interesting nature of multiplex sensors can make an impact. Multiplex sensors use a variety and diversity of receptors immobilized on the single transducer platform composed of several electrodes (electrode array) to simultaneously detect a range of biological analytes taken from one sample. A screen-printed electrode array formed by several sensing electrodes is suitable for detecting multiple signals simultaneously, allowing for differential measurement of several analytes in the solution. A great example of the electrochemical multiplexer is Abbott's i-STAT blood tester equipped with cartridges supporting blood gas, chemistry, coagulation and traumatic brain injury (TBI) marker panels. (Datwyler et al., Patent WO-2018067468-A1) The Abbott i-STAT Alinity multiplex model with TBI Plasma cartridge was FDA-cleared via the 510(k) pathway in January 2021 (https://www.accessdata.fda.gov/cdrh_docs/reviews/K201778.pdf). The cartridge measures two TBI biomarkers (GFAP and UCH-L1) amperometrically using gold working electrodes and an Ag/AgCl reference electrode fabricated on a silicon substrate. (Datwyler et al., Patent WO-2018067468-A1) The i-STAT provides results 15 min after loading a plasma sample onto the cartridge, allowing for the test to be used clinically to assist in determining the need for a head CT scan (a positive result requires a head CT scan while a negative result rules out the need for a head CT). Figure 1 shows both Abbott's iSTAT electrochemical readers.

The key to multiplex testing lies in its functional simplicity; the multiplex sensor can in theory run a multitude of selective and specific tests on a single platform using only a single sample while simultaneously remaining low-cost and highly portable (14, 17–19). Hence, how can a multiplex assay be useful in early screening and diagnostics of BC? BC is a diverse disease with various subtypes, genetic mutations, and DNA damage (20). For analytical scientists, BC is aptly categorized into five immunohistochemical subtypes based on the presence of human receptor tyrosine-protein kinase erbB-2 (HER2), antigen Ki-67, estrogen receptor (ER), and progesterone receptor (PR) (14, 21). These each contribute to the immunohistochemical subtypes: luminal A, luminal B (HER-2 negative), luminal B (HER-2 positive), over-expressed HER-2, and triple-negative BC (21, 22). Additionally, other forms of disease classification consider the stage-dependent biomarkers of BC and look to understand the overexpression of biomolecule-based biomarkers (23).

Within the stage-dependent group, there are diagnostic (healthy vs. BC) (24), prognostic (early BC vs. advanced BC) (25), predictive (providing information regarding the treatment success probability) (25), and therapeutic biomarkers (a target biomolecule for the therapeutic modalities) (26). Biomolecule-based biomarkers are widely considered to be more apt targets for early diagnostics than prognostic or therapeutic biomarkers; however, there is a connection between the prognostic and diagnostic biomarkers which can be leveraged when designing a new BC diagnostic assay (18). The connection between these biomarkers can be used for practical applications. For example, prognostic and predictive biomarkers are two categories that can be linked to therapy success in breast cancer subtypes. After the diagnosis of invasive breast cancer, decisions as to the choice of chemotherapy and adjuvant therapy use. These decisions can be aided by the analysis of prognostic and predictive biomarkers. The molecular markers such as estrogen/progesterone receptors (ER, PR), HER2, and the Mib1/Ki-67 proliferation index are each evaluated and play a role in the standard care of all primary, recurrent, and metastatic BC patients. CEA and CA15-3 antigens are important serum markers. Determining the series of these markers helps to monitor response to the treatment and early detection of recurrence or metastasis. Finally, BC biomarkers are also grouped as genomic (DNA), proteomic (protein-based molecules), transcriptomic (messenger RNA, micro-RNAs), and metabolomic (metabolites) based on their origin, function, and structure (24). Therefore, together with the confirmation of BC, identifying the above-listed subcategories of BC would also represent an upgrade in breast cancer diagnostics and thus subsequent care.

A strategy to improve BC identification and differential diagnosis can be achieved by including additional biomarkers in the current practice. This calls for a diversity of multiplex analysis at a wide scope of molecular levels in contrast to conventional techniques. In this context, new miniaturized biosensing point-of-care (POC) prototypes are under development for fast multiplex monitoring of different biochemical and genetic pathways potentially associated with BC. There are several innovative electroanalytical platforms reported in the literature that are capable of multi-biomarker panel profiling (17, 27).

## 2 Research progress in review until the year 2018

The research aimed towards the development of multiplex electrochemical sensing devices for simultaneous sensing of BC markers published up until 2018 is summarized by Nasrollahpour et al. (published in 2022) (27). This review article focused on the analysis of all types of BC electrochemical sensing approaches (that are for a single-analyte BC biomarker) and highlights the importance of nanomaterials in the design of efficient electrochemical monitoring systems. Although some multiplex electrochemical assays for BC are mentioned in this article (early work only), this technology is developing at a rapid pace. The authors of this review demonstrate the importance of nanostructured electrode components used in biosensor platforms, which allows for full utilization of the sensor's active surface area, leading to an improvement of the sensing signal and the sensor's overall performance. Lakhera and co-workers (14) in their recent review articles (year 2022) are specifically focused on the use of carbon electrodes (screen printed, glassy carbon) and their role as an affordable platform for detection of diverse BC markers (and potential for deployment towards their mass production for POC devices). The work presented here builds upon the analysis of a single-marker biosensor and multiplex BC electrochemical sensors are summarized with a focus on carbon electrodes used as the sensor panel.

Herein, we analyze in detail all research on the development of electrochemical multiplex (duplex, triplex, and quadruplex) BC biomarker sensing platforms. We strive to find the best approach in a multiplex design and to compare: (1) biorecognition elements that are antibody-based (immunosensors) with the nucleic acid aptamers (aptasensors); (2) the type of redox responder and its location (redox tag attached to the sensor probe or target analyte molecule) vs. redox active solutions (e.g., ferri/ferrous cyanides); (3) direct against indirect sensing mechanisms (e.g., use of sandwich-like design with/without enzymatic labels); (4) sensing signal amplification techniques; and finally (5) the reported statistical analysis and the validation of the newly developed electrochemical BC multiplex against the dogmas in BC biomarker assays.

### 2.1 Duplex

**Figure 2:**
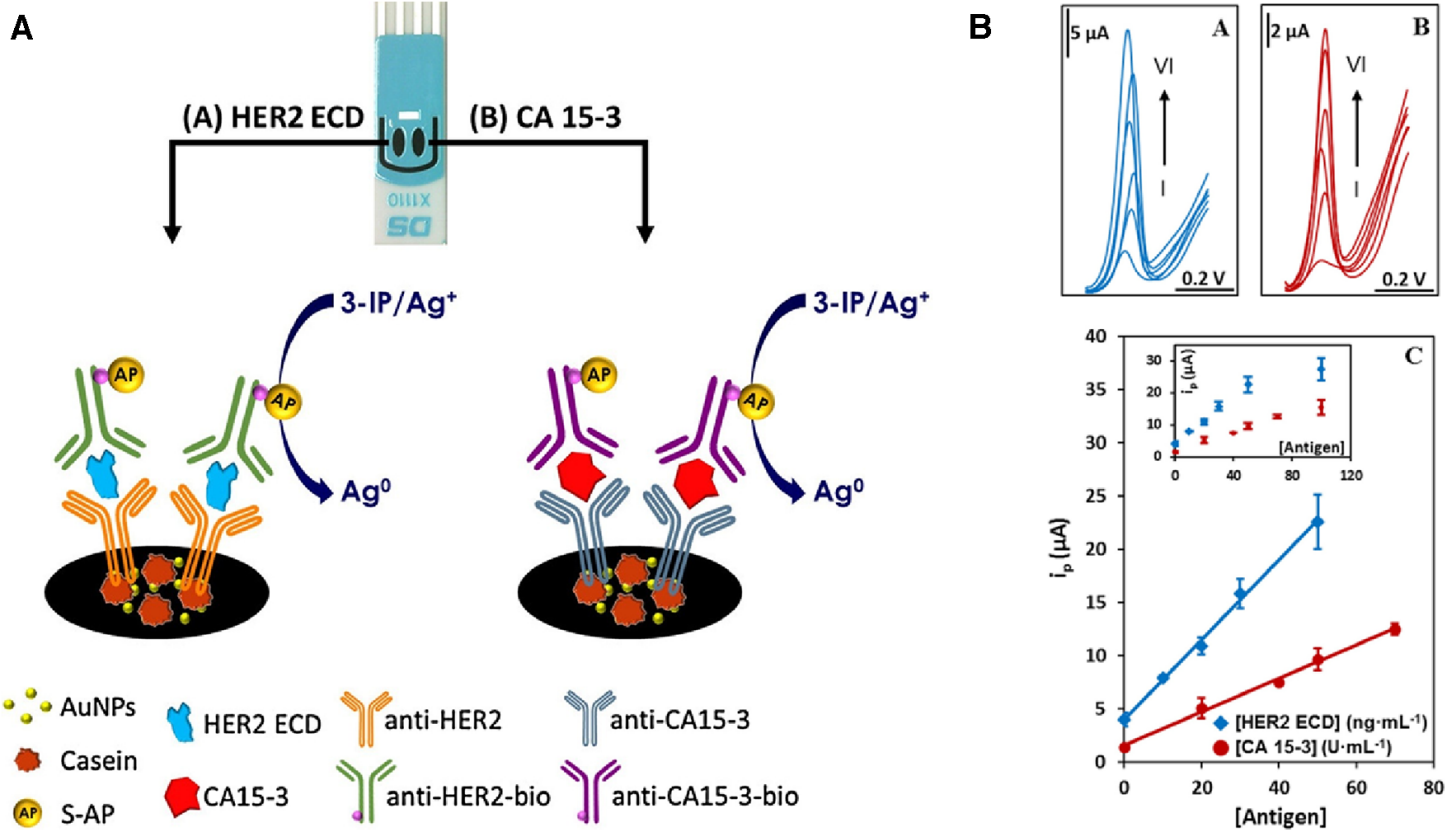
(**A**) An electrochemical dual sandwich-type immunosensor for detection of HER2-ECD and CA 15-3 on Au nanoparticles (NPs)-coated carbon screen-printed electrodes modified with a primary antibody (anti-HER2 and anti-CA15-3) and the complementary detection antibodies (anti-HER2-bio and antiCA15-3-bio) with alkaline phosphatase (AP) as label exposed to a mixture of 3-indoxyl phosphate with silver ions (3-IP/Ag^+^). (**B**) Linear sweep voltammograms for the analysis of HER2-ECD and CA 15-3; with HER2 concentration labelled from I to VI corresponding to 0, 10, 20, 30, 50, 100 ng/ml; and for CA 15-3 at I to VI corresponding to 0, 20, 40, 50, 70, 100 U/ml. The calibration curves built from signals displayed in an inset are shown at the bottom (28). R. Marques et al., Voltammetric immunosensor for the simultaneous analysis of the breast cancer biomarkers CA 15-3 and HER2-ECD, Copyright 2018, with permission from Elsevier.

Cancer antigen 15-3 (CA 15-3) and HER2 extracellular domain (HER2-ECD) are independent biomarkers that when combined, can play a key role in high-risk BC diagnosis. For this reason, Marques and co-workers (28) developed a voltammetric sandwich-type immunosensor for the simultaneous determination of CA 15-3 and HER2-ECD. Dual screen-printed carbon electrodes (made via the printing of carbon on a ceramic platform) were coated with gold nanoparticles, followed by immobilization of captured antibodies by adsorption on each electrode (anti-CA 15-3, and anti-HER2-ECD), and backfilled by casein to minimize non-specific binding. Furthermore, a bi-immunosensor was incubated with a solution containing the antigens (HER2-ECD and CA 15-3) followed by a casting medium with anti-CA15-3- and anti-HER2- bio-antibodies. In such sandwich-type immunoassays, the antigen-antibody interaction is sensed using alkaline phosphatase (AP) as a label on detection antibodies in a mixture of 3-indoxyl phosphate with silver ions (3-IP/Ag^+^). The electrical signal in this system is the peak current intensity associated with the electrochemical activity of silver. This signal is captured by using linear sweep voltammetry, where the potential range is applied to the sensing platform to reach the standard electrochemical potential of silver redox probe (E^0^ of Ag^+^ + e^−^ = Ag^0^), and the current associated with electron (e^−^) flow in this reaction is recorded. The maximum rate of this reaction is represented by a peak current on a voltammogram. The sensor signal results from metallic silver deposition on two individual electrodes (each has a separate electrical connection with the electrochemical reader), and it therefore avoids unwanted overlap of signals. This particular platform design uses the same redox label for the detection of both biomarkers (Figure 2). A sensor platform was inserted into an electrical connector, allowing for interfacing between working electrodes and a dual-channel electrochemical reader (bi-potentiostat) (28). The reported LODs for the selected biomarkers were 5.0 U/ml for CA 15-3 and 2.9 ng/ml for HER2-ECD, which are comparable to the conventional assay measured by ELISA with sensitivity within 0–240 U/ml (Creative Diagnostic, Catalog # DEIA2145) and 10^−12^–10^−9^ g/ml (Bio-Techne, Catalog # DHER20) for CA 15-3 and HER2-ECD, respectively.

**Figure 3:**
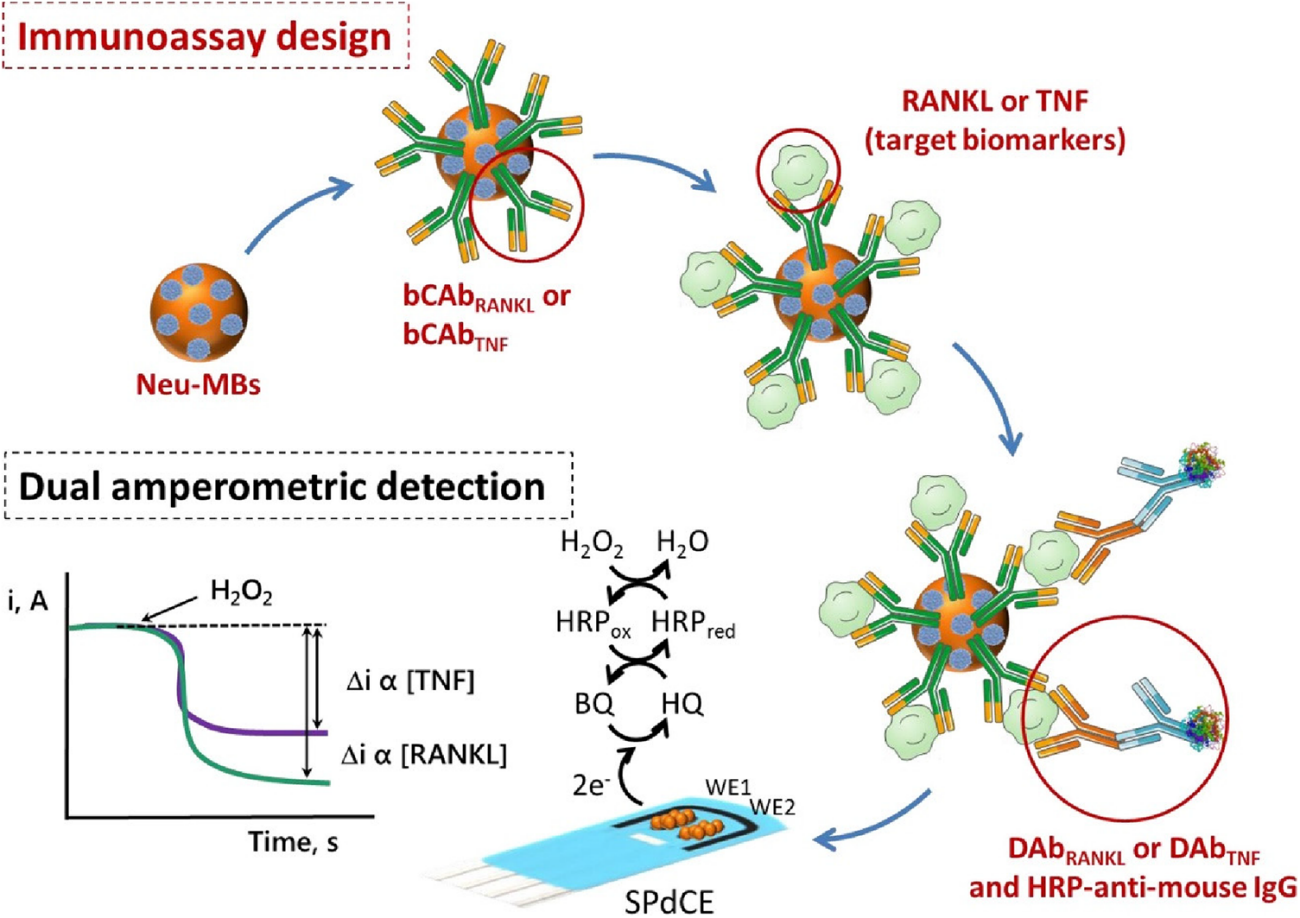
An electrochemical immunosensor designed on carbon screen-printed electrode (sPdCE) for the dual amperometric determination of RANKL and TNF biomarkers (29). The steps undertaken in prototyping the platform include (from the top left) immobilization of biotinylated capture antibodies (bCAbRANKL or bCAbTNF) onto neutravidin-functionalized magnetic microbeads (Neu-MBs) immobilized on working electrode 1 (WE1) and 2 (WE2), respectively followed by capturing RANKL and TNF on WE1 and WE2, the attachment of HRP-labeled secondary antibodies (HRP-anti-mouse-IgG) to WE1 and WE2 to form a sandwich-type immunocomplex (bottom right); and recording electrical signal (i, A) generated by the HRP reduction of H_2_O_2_ mediated by HQ (bottom left) (29), Alejandro Valverde et al., Electrochemical immunoplatform to improve the reliability of breast cancer diagnosis through the simultaneous determination of RANKL and TNF in serum, Copyright 2020, with permission from Elsevier.

The simultaneous determination of Tumor Necrosis Factor-alpha (TNF-α), and Receptor Activator of Nuclear Factor-kB Ligand (RANKL) via a sandwich-type immunosensor built on a dual screen-printed carbon electrode platform was explored by Valverde and co-workers (29). This research group developed an immunoplatform to identify both biomarkers in untreated human serum from BC patients. Like the approach proposed by Marques (28), two carbon working electrodes printed on a ceramic substrate were interfaced via an electrical port with a multichannel electrochemical reader. However, the steps undertaken to engineer this duplex sensor were different from Marques's methodology. Namely, three main assemblies: biotinylated specific capture antibodies, detector antibodies, and HRP (horseradish peroxidase)-labelled secondary antibodies (HRP-anti-mouse IgG), were immobilized onto magnetic microbeads (MBs) (29). A vast portion of work focussed on the preparation and optimization of these bioconjugates. Firstly, neutravidin-functionalized magnetic beads (Neu-MBs) were functionalized by incubation with RANKL and TNF biotinylated capture antibody (bCAbRANKL or bCAbTNF) prepared in a buffer solution. These were then washed with a blocking buffer (BB) solution. Microcentrifuge tubes containing bCAbRANKL-MBs were then placed in an incubator shaker with a mixture containing RANKL standards (or the sample to be analyzed), RANKL detector antibody (DAbRANKL), and HRP-anti-mouse IgG prepared in the BB solution. For the TNF-sensing bioconjugates, bCAbTNF-MBs were incubated with TNF standards (or the sample to be analyzed) and prepared in BB solution. Next, the DAbTNF-TNF-bCAbTNF-MBs were washed, and subsequently incubated with HRP-anti-mouse IgG prepared in BB solution. Finally, the modified MBs were washed and left as a suspension in a phosphate buffer solution. Such MBs-modified bioconjugates were drop-cast onto the carbon working electrodes, WE (HRP-anti-mouse IgG-DAbRANKL-RANKL-bCAbRANKL-MBs onto WE1 and HRP-anti-mouse IgG-DAbTNF-TNF-bCAbTNF-MBs onto WE2). To enhance the attachment of MB-immunoconjugates to working electrodes, the electrochemical platform was placed in the polymer casing with encapsulated neodymium magnets. This magnetic field-assisted sensor platform was subsequently immersed into a buffer solution containing hydroquinone (HQ) and H_2_O_2_ in an electrochemical cell. An electrical signal was acquired using amperometry, where the constant potential is applied to the working electrode and the current generated during the electrochemical process was recorded and plotted vs. time. In this sensing process, the current is generated by the reduction of H_2_O_2_ using HRP mediated by HQ (Figure 3). This amperometric dual platform showed a detection limit of 3.0 pg/ml and 2.6 pg/ml for TNF and RANKL, respectively (29). For reference, the sensitivity of ELISA tests sold by Thermo Fisher Scientific for TNF and RANKL are 1.7 pg/ml (Catalog # KHC 3,011) and 9.38 pg/ml (Catalog # EEL071), respectively.

Another sandwich-type electrochemical biosensor was prototyped to detect protein MUC-1 biomarkers and MCF-7 BC cells—in which MUC-1 is overexpressed (30). The concept of this duplex sensor significantly differs from the work presented in the aforementioned sections. The main differences are (1) the use of a glassy carbon disk electrode instead of screen-printed electrodes, and (2) also subsequent measurement of each biomarker (MUC-1 protein) and MUC-1 positive BC cells. For this, each electrode is connected to a single channel electrochemical reader (meaning that the sensing signal is not acquired simultaneously—as seen in previous works) (28, 29), Lastly, the electrode composition/design was the same for both markers (no additional modification of the electrode was applied to accommodate binding of pure MUC-1 protein vs. whole MCF-7 cells). The sensor was fabricated using four subsequently immobilized assemblies, which are the MUC-1 antibody covalently attached to graphene (deposited onto glassy carbon disk), MUC-1 analyte (or MUC-1 positive cells), MUC-1 binding aptamer connected to 12-mer polycytosine (C_12_) used as a template for Ag nanoclusters (NCs), and metallic silver (redox responder and signal amplifier) deposited on the top of hairpin structures for amplification of electrical signal.

**Figure 4:**
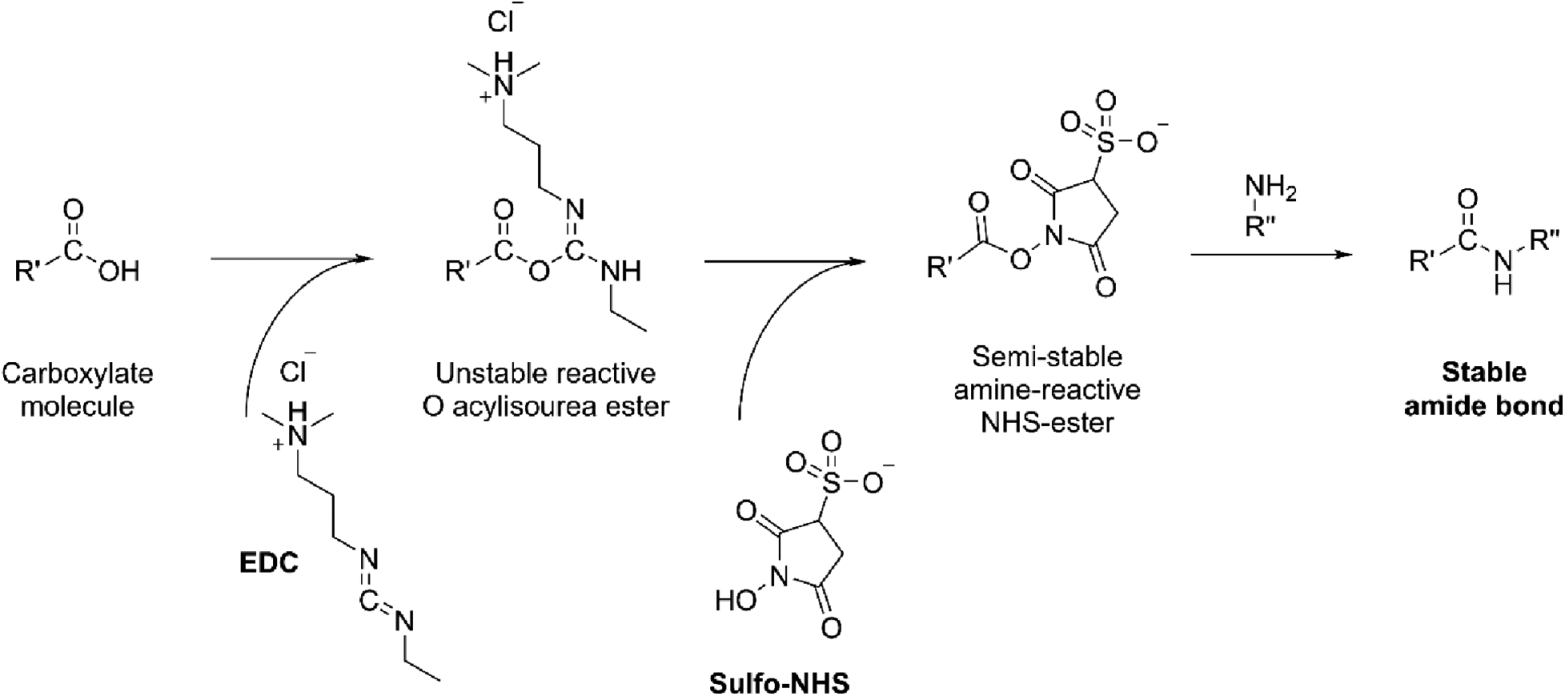
The formation of amide bond from the carboxylate moiety on the carbon with amine (NH_2_-R”) present in antibody catalyzed by EDC/NHS.

In brief, the sensor assembly starts with the coating of graphene/MUC-1 antibody bioconjugate on the glassy carbon disk. This step ensures sufficient surface area needed to accommodate antibodies (bare glassy carbon is a mirror-polished flat surface that is not suitable for any chemical modification needed to attach antibodies). For this reason, graphene was first mixed with EDC [N-ethyl-N'-(3-dimethylaminopropyl)carbodiimide] and NHS (N-Hydroxysuccinimide), and then with an anti-MUC-1 antibody to form an amide-type bond from amino-groups (present in antibody) and carboxyl moieties in graphene (Figure 4).

Afterwards, graphene-anti-MUC-1 was drop-cast onto the glassy carbon, dried, and exposed to analyte MUC-1 (or MUC-1 positive BC cells) for incubation (1 h). In parallel, the MUC-1 binding aptamer connected to 12-mer polycytosine (C_12_) was used as a template for Ag nanocluster (NCs) synthesis. Here, the researchers took advantage of the high affinity of Ag^+^ to negatively charged nucleic acids (from an array of phosphate groups) to control the growth and morphology of Ag NCs. The concept of using biopolymers as templating agents is particularly appealing due to the simplicity of integration of Ag nanostructures within the DNA's template (C_12_). The presence of four complementary bases (CGCG) at the ends of 12-mer polycytosine (C_12_) creates a stem-loop structure with a long C-loop (CGCGC_12_). The synthesis of silver nanostructures under CGCGC_12_ protection yields a well-designed Ag deposit on one end of this modified aptamer. In this work, the DNA strands contain MUC1 aptamer and C_12_ template (5′ GCAGTTGATCCTTTGGATACCCTGGC_12_-3′). The hairpin MUC-1 aptamer-Ag NCs were drop cast onto the MUC-1 (or MCF-7 cell) coated platform (1 h of incubation) and washed to remove unbound MUC-1 aptamer-Ag NCs. This assembly rules on specific MUC-1 biomarker-aptamer binding.

At this stage of sensor development, a square wave voltammogram (SWV) was recorded and the electrical signal corresponded to the reduction of silver originating from Ag NCs. Similar to the voltammetry method used by Marques et al. (28), this electrochemical response was initiated by applying the potential reaching the standard reduction potential of Ag and recording the current (flow of e^−^) needed to reduce Ag ions. Since the Ag signal was weak, an additional silver enhancement step was applied to amplify the electrochemical response of Ag. This was carried out by immersing the sensor into an aqueous solution of AgNO_3_ followed by the reduction of Ag^+^ using NaBH_4_. The SWV spectra were then recorded with the same electrochemical settings and a significantly higher current from the Ag responder was observed. The immunosensors were then tested at different concentrations of MUC1 (or MCF-7 cells), and the SWV peak current increased linearly with increasing amounts of analyte. At higher concentrations of MUC1, more Ag NCs were deposited onto the electrode due to binding between the aptamer and MUC1. With more Ag NCs on the electrode serving as the nucleus, the deposited silver enhancer/primary redox responder is increased and thus is the corresponding current generated by Ag ions. This additional silver enhancement improves the sensitivity of the immunosensors approximately 20 times over. This enabled the detection of MUC1 at 0.5 nM, which was significantly lower than LODs reported by other groups that tested electrochemical and optical biosensors for the same BC biomarkers (30). As an important step forward, this group also reported LODs for their platform tested in the presence of interfering proteins such as carcinoembryonic antigen (CEA), α-fetoprotein (AFP), human IgG and human serum albumin (HSA) and it was found that the signal variation due to interfering proteins was less than 10% of that without interfering proteins. The same sensor validation was carried out for MUC-1-negative cells (HepG2 cells or HCT116 cells) instead of MCF-7, and only weak current signals were noted. This demonstrates a low affinity between the sensor and non-specific cancer cells.

The sensor response was monitored by square wave voltammetry and LODs were reported as 0.5 nM, and 50 cells/ml for MUC-1 and MCF-7, respectively (30). For reference, a standard MUC-1 ELISA test has a detection range from 4.1 mU/ml to 1,000 mU/ml (Thermo Fisher Scientific, Catalog # EHMUC1).

The same target cancer cells (MCF-7) and MUC-1 protein biomarkers were detected using silver-labelled biomolecules and a poly(glutamic acid)/carbon nanotube nanocomposite. MUC-1-binding aptamer was attached to the glassy carbon electrode and then modified with poly(glutamic acid)/carbon nanotube nanocomposite. This was followed by the immobilization of another Ag nanoparticle-labelled aptamer for recognition of MCF-7 cells. Such a sandwich design with an aptamer-cell-aptamer architecture has some similarities to the dual sandwich biosensor reported by Guo *et. al (*30) as detailed in the previous section. For example, both biosensors were constructed on a single glassy carbon electrode disk and the same molecular design was used to detect both the MUC-1 protein and the whole MUC-1-positive cell. A single-channel electrochemical reader was used to collect electrochemical signals separately for MUC-1 and MCF-7 cells. Two types of aptamers were used in this design, one was a binding aptamer covalently attached to the polymer-coated disk electrode and the second was silver nanoparticle (AgNPs)-labelled aptamer (detection aptamer) that contained the redox responder (Ag nanoparticles). The main difference between this work and the concept proposed by Guo and colleagues is the method of attaching the aptamer to the disk electrode. Namely, glassy carbon was coated with a layer of multiwalled carbon nanotubes (MWCNTs), whose role is to enhance electron conductivity within the poly(glutamic acid), PGA polymer. PGA contains numerous carboxylic functional groups and therefore it is a suitable reactant for the 5′-NH_2_-terminal ends of synthetic aptamers. The connection between the two was initiated by EDC/NHS as demonstrated in Figure 4. Afterwards, MCF-7 cells, or MUC-1 proteins were cast on the electrode and captured through the specific interaction between the aptamer and the target. As the last step completing the sandwich design, the electrode was incubated for 40 min with (AgNPs)-labelled MUC-1 aptamer [detection aptamer; Ag is modified first with reduced glutathione (GHS) that binds with the aptamer], is subsequently washed, and is finally subjected to electrochemical testing.

In this biosensor design, Ag acts as a redox responder (similar to work by Guo et al.) (30). However, different electrochemical technique was used to quantify Ag as a signalling probe. Specifically, in this work, the anodic stripping voltammetry of AgNPs was evaluated for quantification of MCF-7 cells. An electrode was exposed to an acid to dissolve AgNPs. Then, differential pulse anodic stripping voltammetry was performed for the quantification of released Ag^+^ from the complex. In anodic stripping voltammetry, the analyte of interest is first collected on the working electrode during a deposition step for a known time and stripped from the surface into the solution by applying an oxidizing potential. The electrochemical signal of Ag oxidation was proportional to the concentration of Ag ions in the solution. The quantity of realized Ag ions corresponded to the amount of captured MCF-7 cells or MUC-1 protein. This research team also used a secondary redox responder in the solution, [Fe(CN)_6_]^3−/4−^, to display the change in resistivity of the layer-by-layer assembly. As such, an increased concentration of bioconjugates attached to the glassy carbon corresponds to higher resistance of the sensor platform. This consequently causes smaller electrochemical signals for Fe^2+^ ↔ Fe^3+^ conversion in the [Fe(CN)_6_]^3−/4−^ redox responder. This is due to the less conductive nature of the bioconjugates, therefore making the sensor electrode more resistant to current flow.

This prototype demonstrated a limit of detection at 3 ng/ml and 25 cells/ml for MUC-1 and MCF-7, respectively (31). The bio-electrode design proposed here is simpler than the sensor developed by Guo et al. for the same target analytes and the LODs for both markers seem to fall within a similar range. Yet, comparing these LODs with the commercial ELISA cell counter kit mentioned above, the proposed sensing platform for MUC-1 protein and MCF-7 cells should be further improved so that their sensitivity is similar to conventional assays.

**Figure 5:**
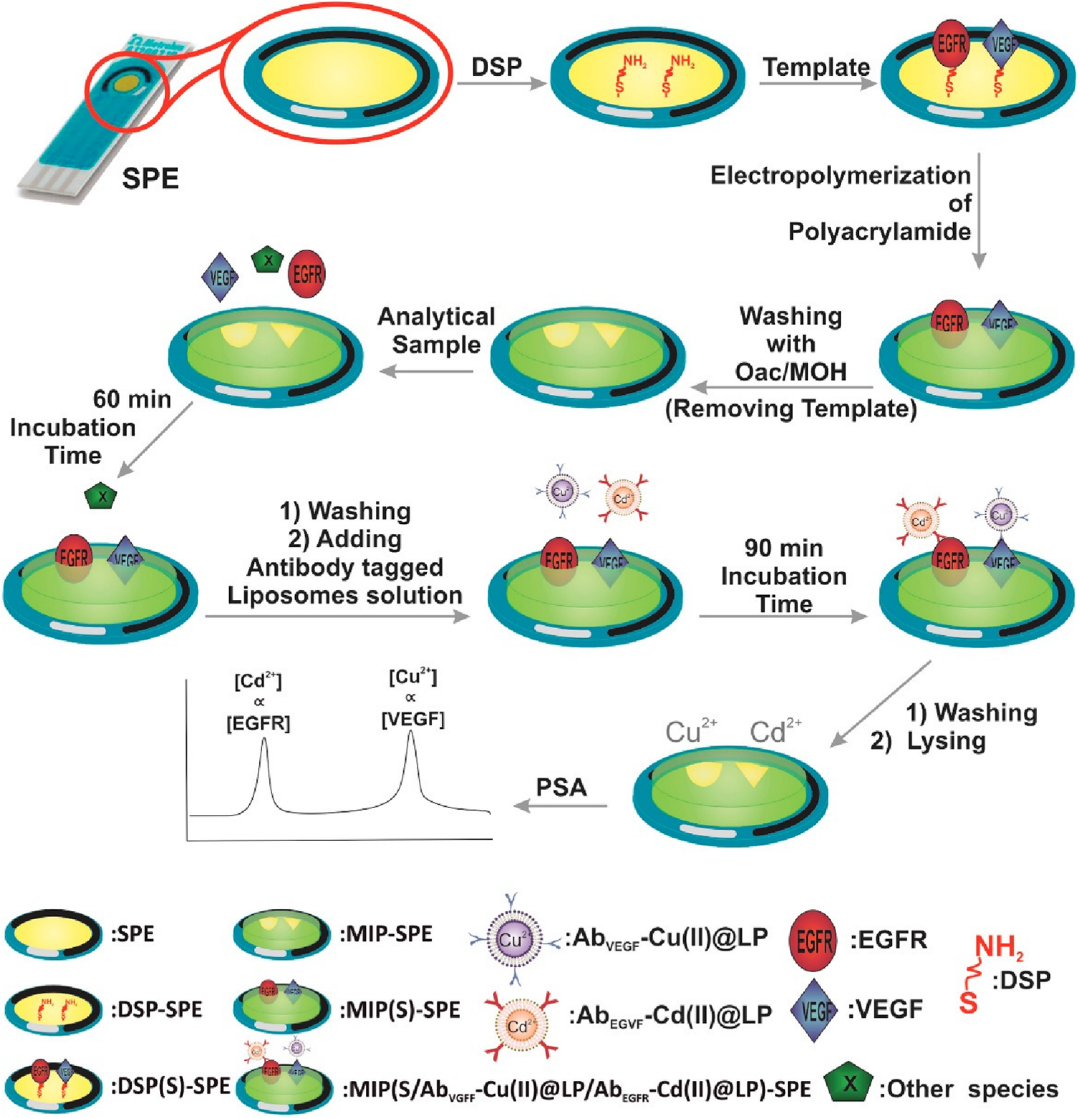
An electrochemical duplex designed on a single gold screen-printed electrode for simultaneous detection of EGFR and VEGF BC biomarkers (32). M. Johari-Ahar et al., Development of a molecularly imprinted polymer tailored on disposable screen-printed electrodes for dual detection of EGFR and VEGF using nano-liposomal amplification strategy, Copyright 2018, with permission from Elsevier.

Johari et al. designed an electrochemical biosensor platform made of a molecularly imprinted polymer that responded to epidermal growth factor receptor (EGFR) and vascular endothelial growth factor (VEGF) in serum samples (32). Their sensing platform was built on a gold screen-printed electrode modified with 3,3′-dithiodipropionic acid di(N-hydroxysuccinimide ester), while the EGFR and VEGF proteins were covalently attached to the electrode. Subsequently, nano-liposomes containing metal ions Cd^2+^ and Cu^2+^ were decorated with antibodies specific for EGFR and VEGF, and potentiometric striping analysis was used for indirect quantification. The steps taken during the fabrication of this biosensor platform are demonstrated in Figure 5.

The first step was to modify the gold surface with thiol self-assembled monolayers (SAMs) to create a chemical bond (linker) with the gold surface at one end, and the -NH_2_ motifs needed for further conjugation with the target analyte molecule at the opposite end of SAMs. Afterwards, the platform was incubated with EGFR and VEGF proteins to form a covalent bond on the electrode surface via amide bond formation (Figure 4). Furthermore, molecularly imprinted polymer (MIP) was synthesized from acrylamide and N,N′-methylene-bis(acrylamide) around the EGFR and VEGF template. After polymerization was completed EGRF and VEGF templates were removed from the platform using oxalic acid (OXA). On one side, Cu(II) and Cd(II)-functionalized liposomes (Cu(II)@LP and Cd(II)@LP) were synthesized following an established protocol and resulted in nano-sized conjugates that were further modified with antibodies (AbEGFR and AbVEGF). These immunoconjugates were formed during several chemical steps which included a sulfo-SMCC crosslinking with liposomal' sulfhydryl moieties. This was followed by a washing and column separation of unbound antibodies from the antibody-tagged LPs (Cd(II)@LP and Cu(II)@LP). To assess the sensor, the MIP-SPE platform was immersed in standard solutions of VEGF and EGFR proteins, washed and incubated with AbEGFR-Cd(II)@LP and AbVEGF-Cu(II)@LP. The redox responders were Cd^2+^ and Cu^2+^ ions, and the concentration was measured by potentiometric stripping analysis (PSA) that was further correlated with EGFR and VEGF encapsulated within the MIP network. The electrochemical signal was generated using a constant current (flow of e^−^) through the electrode, resulting in the electrochemical reduction of cations: Cu^2+^ + 2e^−^ ↔ Cu^0^ and Cd^2+^ + 2e^−^ ↔ Cd^0^. The potential change associated with the electrochemical reduction of metal ions was measured (dt/dE). This indirect electrochemical test has a similar concept to biosensors discussed in the preceding section.

The set of experiments reported by this group was carried out in a standard solution of biomarkers, serum samples, and whole blood samples, and demonstrated promising selectivity and reported LODs of 0.01 and 0.005 pg/ml for EGFR and VEGF, respectively (32). When compared to commercial standards, Invitrogen's EGFR (Full-length) Human ELISA Kit reports an assay range of 0.31–20 ng/ml (Thermo Fisher Scientific, Catalog # KHR9061). For VEGF measured in serum, EDTA plasma, heparin plasma, and citrate plasma, the assay range is 31.3–2,000 pg/ml (Bio-Techne, Catalog # DVE00).

### 2.2 Triplex & quadruplex

**Figure 6:**
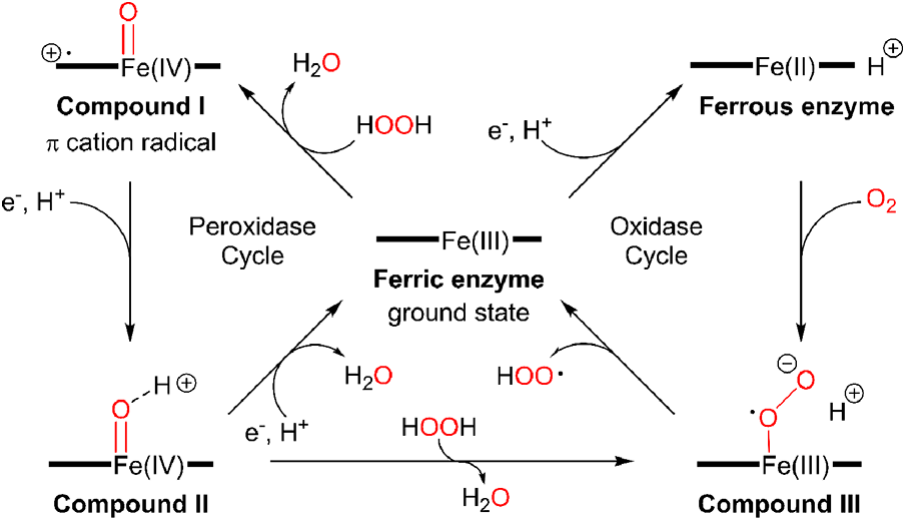
A mechanism of electrochemical activity of HPR. Reproduced with permission from The catalytic pathway of horseradish peroxidase at high resolution by G. I. Berglund et al. (34) Copyright 2002 Springer Nature.

**Figure 7:**
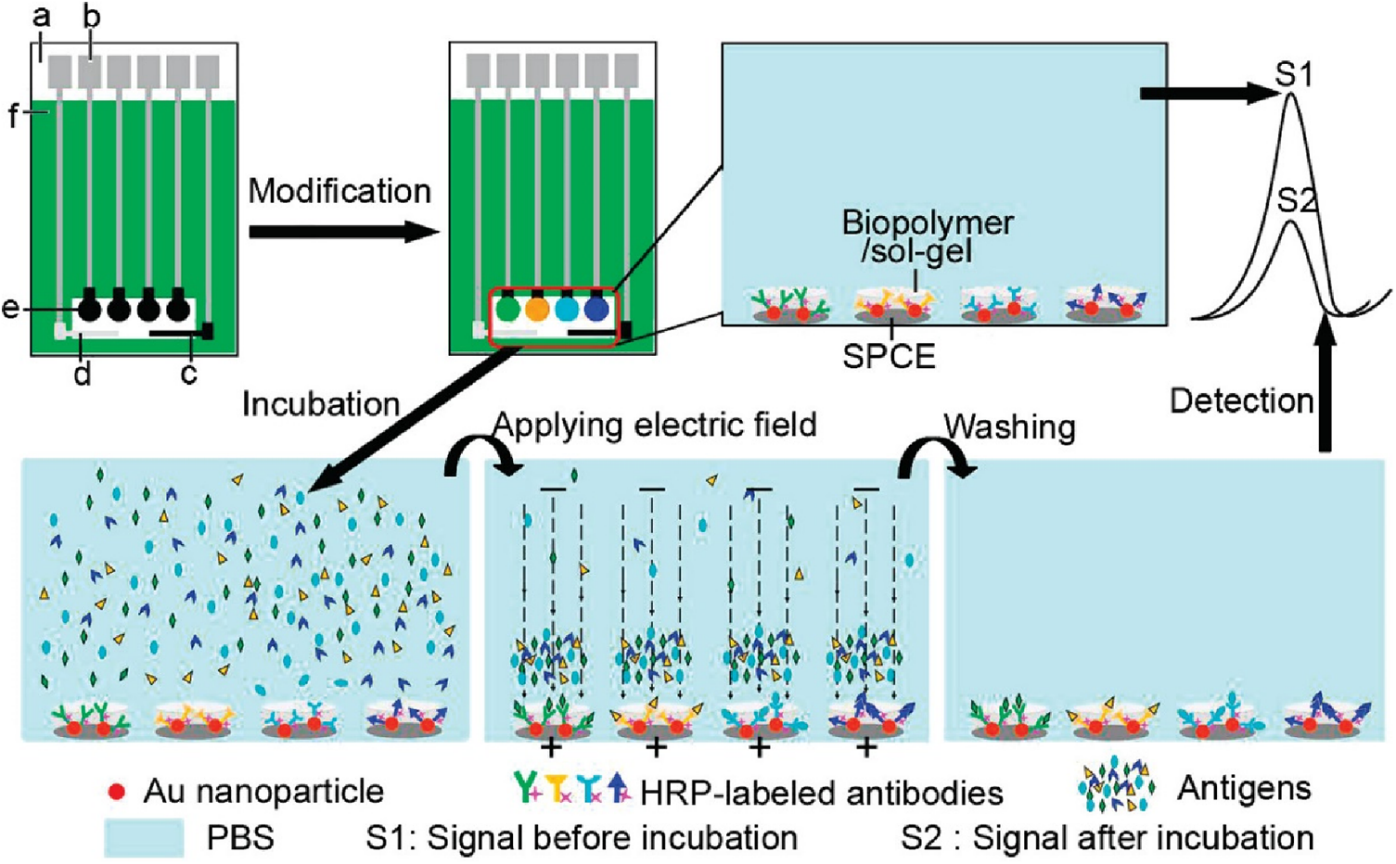
Electrochemical immunosensor array (ECIA) with an electric field-driven incubation process for simultaneous detection of CA 153, CA 125, CA 199, and (CEA). (a) nylon sheet, (b) silver ink, (c) graphite auxiliary electrode, (d) Ag/AgCl reference electrode, (e) graphite working electrode, and (f) insulating dielectric. Adapted with permission from Electric Field-Driven Strategy for Multiplexed Detection of Protein Biomarkers Using a Disposable Reagentless Electrochemical Immunosensor Array by Jie Wu et al. (33) Copyright 2008 American Chemical Society.

There is limited work done for both triplex and quadruplex BC biosensors. Pioneering research on the simultaneous detection of carbohydrate antigens 153, 125, and 199 (CA 153, CA 125, CA 199) and carcinoembryonic antigen (CEA) markers was published by Wu and co-workers in 2008 (33). Authors demonstrated a disposable array of four graphite working electrodes printed on a single panel (Figure 7). All working electrodes shared the same Ag/AgCl reference and graphite auxiliary electrodes. The approach proposed here allows for (1) miniaturization of the platform and (2) reduction of inconsistency that can often originate from the flawed print of reference and counter electrode. This prototype used a four-channel potentiostat (each interfaced with an individual working electrode) allowing for the simultaneous measurement. Commercial HRP (horseradish peroxidase)-labelled CA 153, CA 125, CA 199, and CEA mouse monoclonal antibodies were mixed with gold nanoparticles, biopolymer containing chitosan, 3-(aminopropyl) triethoxysilane, and tetraethoxysilane. In this case, metallic gold nanoparticles were not used as a redox responder—their function was to improve the electron transfer within the bioconjugate and therefore facilitate HRP electroactivity. HRP acts as the redox probe whose signal is recorded in this prototype. The biopolymer was used to fix the antibody onto the carbon working electrodes. Before use, the sensor was incubated with 1% bovine serum albumin to prevent nonspecific adsorption. Before testing the prototype against target analytes (CA 153, CA 125, CA 199, and CEA), the baseline voltammogram is recorded. The sensor pre-polarization was done by applying electrochemical potential from the range that covers the redox activity of HRP, and subsequent immersion in the analyte solution. The electrochemical responses were derived from the direct electroactivity of Fe(III) ↔ Fe(II) redox centre immobilized within the HRP molecule (Figure 6).

It is important to note that the sensing event was enhanced by applying a driving potential, which produces an electric field at the electrode/electrolyte interface. This external electrophoretic force accelerates the transport of charged antigen molecules to electrodes and thus accelerates the binding of biomarkers. With an increasing amount of attached analyte, the immunocomplex blocks the direct electron transfer between HRP and the electrode. This was recorded as an increase in the total impedance of the sensor and corresponded to the decrease in recorded current projected on voltammograms. An increase in the concentration of proteins attached to the electrode surface caused a quenching of the electrochemical signal associated with the HRP's redox activity. This electrochemical signal was generated directly from the HRP centre without the help of any electron transfer mediator or enzymatic substrate (Figure 7).

The quadruplex platform showed detection limits of 0.04, 0.06, 0.1, and 0.03 U/ml for CEA, CA15-3, CA-19-9, and CA-125, respectively (33). Reported LODs of corresponding commercial assays are as follows: CA15-3 can be detected in the range of 0–240 U/ml (as mentioned above), 0.3–200 U/ml for CA-19-9 (Ray Biotech, Human CA19-9 ELISA Kit), 0.6–400 U/ml for CA-125 (Ray Biotech, Human CA-125 ELISA Kit), and 0.343–250 ng/ml for CEA (Thermo Fisher Scientific, CEA Human ELISA Kit Catalog # EHCEA).

Finally, an electrochemical triplex biosensor for simultaneous identification of CA-125, CA-15-3, and CEA was constructed by immobilizing capture antibody (Ab1) on screen-printed carbon electrodes (35). The sensor surface was further modified with platinum particles to label the secondary antibody (Ab2). This step catalyzes the electro-reduction of H_2_O_2_, which produces detectable signals for the readout of analytes. Table 1 provides a summary of electrochemical multiplex biosensors targeting BC biomarkers.

**Table 1:** An overview of the electrochemical multiplex platforms targeting BC serum markers.

Year	Platform	BC Markers	LOD	Ref
2015	Duplex	MUC-1; MCF-7	0.5 nM; 50 cells/ml	(30)
2018	Duplex	CA15-3; HER2-ECD	5 U/ml; 2.9 ng/ml	(28)
2018	Duplex	MUC-1; MCF-7	3 ng/mL; 25 cells/mL	(31)
2018	Duplex	EGFR; VEGF	0.01 pg/ml; 0.005 pg/ml	(32)
2020	Duplex	TNF; RANKL	3 pg/ml; 2.6 pg/ml	(29)
2020	Duplex	CD24; CD340	1.94 × 10^5^ exosomes/ul; 1.02 × 10^6^ exosomes/ul	(44)
2021	Duplex	EGFR; ICAM-1	10^3^ to 10^5^ cells	(36)
2021	Duplex	CEA; CA15-3	3.9 pg/ml; 5.8 × 10^−3^ U/ml	(40)
2022	Duplex	EpCAM; HER2-ECD	Single particle/ml	(38)
2022	Duplex	miRNA-21; CA15-3	1.2 fM; 0.14 U/ml	(39)
2014	Triplex	CEA; CA15-3; CA-125	7.0 pg/mL; 0.001 U/ml; 0.002 U/ml	(35)
2020	Triplex	CEA; CA15-3; CA-125	0.04 pg/ml; 0.04 mU/ml; 0.04 mU/ml	(41)
2021	Triplex	miRNA-155; miRNA-21; miRNA-16	9.79 × 10^−16^ M; 3.58 × 10^−15^ M; 2.54 × 10^−16^ M	(42)
2021	Triplex	MUC-1; CA15-3; HER2-ECD	0.53 ng/ml; 0.21 U/ml; 0.50 ng/ml	(43)
2023	Triplex	CD63; HER2; EpCAM	3.4 × 10^3^–3.4 × 10^8^ particles/ml	(45)
2008	Quadruplex	CEA; CA15-3; CA19-9; CA-125	0.04 U/ml; 0.06 U/ml; 0.1 U/m; 0.03 U/ml	(33)

Similar to work reported by Wu et al. (33), the triplex sensor developed herein was made of three carbon screen-printed electrodes that shared the same Ag/AgCl reference and carbon counter electrodes which were coupled with a multichannel electrochemical reader. This reader simultaneously collected electrical signals for each working electrode. The immunosensor was constructed in two steps. Firstly, Pt nanoparticles were synthesized and combined with CEA Ab2, CA125 Ab2 or CA153 secondary antibody Ab2 (detection antibody). These Pt-Ab2 conjugates are proposed to be formed from the interaction between the amino groups of Ab2 and the Pt surface. The carbon working electrodes were prepared by applying a coating of graphene layer. The EDC/NHS was drop-cast to initiate an amidation reaction between the carboxylic groups in graphene and the available amine groups of Ab1 (anti-CA125, anti-CA153 and anti-CEA) according to the reaction mechanism demonstrated in Figure 4. BSA was also cast prior to exposing the sensor to solutions containing target analytes (CA125, CA153 and CEA) to minimize non-specific binding. After washing, secondary antibody (Ab2)-Pt conjugates were deposited on the immunosensors, and residual reactants were removed through a buffer wash. As the final step, the multiplex platform was immersed in a buffer containing H_2_O_2_ and analytes were quantified from signals associated with the electrochemical conversion of hydrogen peroxide. The intensity of peak current corresponding to H_2_O_2_ reduction scales with increasing concentrations of (Ab2)-Pt conjugates. The amount of (Ab2)-Pt was dependent on the amount of CA125, anti-CA153, and anti-CEA markers captured by Ab1 (anti-CA125, anti-CA153 and anti-CEA).

This multiplex device did not contain an enzymatic catalyst or any additional electrochemical redox mediator, it instead took advantage of the strong catalytic activity of platinum (Pt nanoparticles) toward the reduction of H_2_O_2_. The sensitivity of this multiplex platform was improved by the utilization of graphene nanosheets (NSs), which promoted fast electron transfer and expanded the surface area of the electrode, thus allowing for large loading of capture antibodies (Ab1). Taking advantage of high catalytic activity and large surface area of Pt nanoparticles, the reduction of H_2_O_2_ present in measurement solution (external redox responder) is accelerated. The triplex platform was also tested against serum samples containing known concentrations of each of the above markers.

The proposed immunoassay demonstrated very low detection limits at 0.001 U/ml, 0.002 U/ml, and 7.0 pg/ml for CA 15-3, CA-125, and CEA, respectively (35), which all fall below that of the commercial assays mentioned above.

## 3 Recent progress: 2019–2023

This section highlights the most recent developments in electrochemical multiplex sensing of breast cancer biomarkers, with emphasis on the detection of new BC markers and new biomolecular modifications of sensing platforms (Table 1).

### 3.1 Duplex

**Figure 8:**
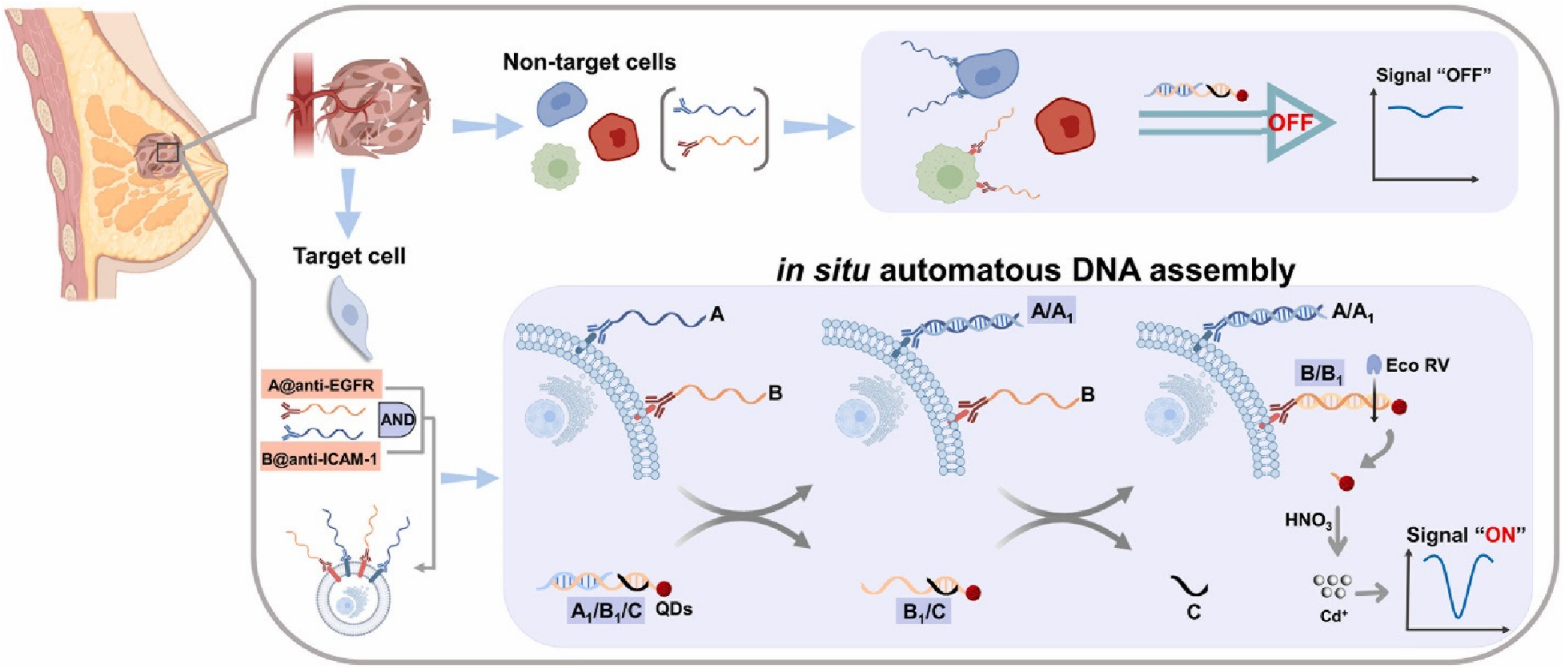
Schematic illustration of *in situ* automatous DNA assembly for the discovery of dual therapeutic targets in breast cancer. Bing Han et al., Identification of dual therapeutic targets assisted by *in situ* automatous DNA assembly for combined therapy in breast cancer (36). Copyright 2021, with permission from Elsevier.

Han et al. were able to prepare a set of DNA capture probes, each linked to one of two antibodies—epidermal growth factor receptor, EGFR or intercellular adhesion molecule-1, ICAM-1 (36). Figure 8 depicts an illustration of in situ automatous DNA assembly for the discovery of dual therapeutic targets in BC. Anti-EGFR and anti-ICAM-1 antibody biorecognition elements were attached to their respective DNA probes. Surface markers EGFR and ICAM-1 would thus be tagged by the antibody and the DNA probes could be targeted via a toe-hold-mediated strand displacement reaction (TSDR)—a displacement reaction occurring at the single-stranded (sticky) end, called a toehold, where the shorter strand of the duplex exchanges with a longer complementary invader strand to synthesize a duplex of DNA (37). This targeted DNA, named B1, is uniquely able to catalyze the synthesis of quantum dots which could be measured via an amplified stripping signal (using anodic stripping voltammetry at a voltage of −1.2 V for 480 s, followed by DPV scanned from −1 V to −0.5 V at 50 mV amplitudes) of the quantum dots from the B1 strands. This indirect measurement reported a detection range of 10^3^–10^5^ cancer cells.

In the work completed by Hashkavayi et al., the researchers looked to fabricate a SPCE-based EpCAM and HER2 exosomal aptasensor with an added dual rolling circle amplification reaction initiator (an in-situ DNA amplification technique) thus allowing for single particle/ml detection limits (38). To do this, a multi-walled carbon nanotube, ionic liquid, and chitosan composite was created and deposited simultaneously with gold nanoparticles to the SPCE and subsequently modified with CD63-, HER2-, and EpCAM-specific aptamers; the latter of the two aptamers was modified with primers to initiate rolling circle amplification upon binding to EpCAM and HER-2 positive exosomes. Thus, the CD63 capture aptamers were used as an exosomal catch-all, and subsequent binding to the HER2 and EpCAM-specific aptamers activated rolling circle amplification could be initiated, thereby generating poly-guanine and poly-thymine repeats. These repeats could then uptake electrochemically active Pb^2+^ and Cu^2+^ ions in solution which could be interpreted at a detection limit of 1 particle/ml using differential pulse voltammetry. If this technique can be made storable for extended periods, it could provide a highly selective and sensitive detection platform for breast cancers.

Pothipor et al. looked to detect CA15-3 and miRNA-21 via the use of a poly(3-aminobenzylamine)/two-dimensional (2D) molybdenum selenide/ graphene oxide nanocomposite modified two-screen-printed carbon electrode array (39). To do this, SPEs were each functionalized with a molybdenum-selenide/graphene oxide and subsequently with 2,3-diaminophenazine-gold nanoparticles and toluidine blue-gold nanoparticles. CA 15-3 protein and miRNA-21 were measured via differential pulse voltammetry (DPV) by measuring immunoreaction and hybridization, respectively, on the electrode surfaces. The measurements, both before and after the reaction, could then be evaluated for proportional changes in signal due to the dampening of redox reaction signals of select redox dyes in solution. LODs were reported to be 1.2 fM and 0.14 U/ml for miRNA-21 and CA 15-3, respectively.

**Figure 9:**
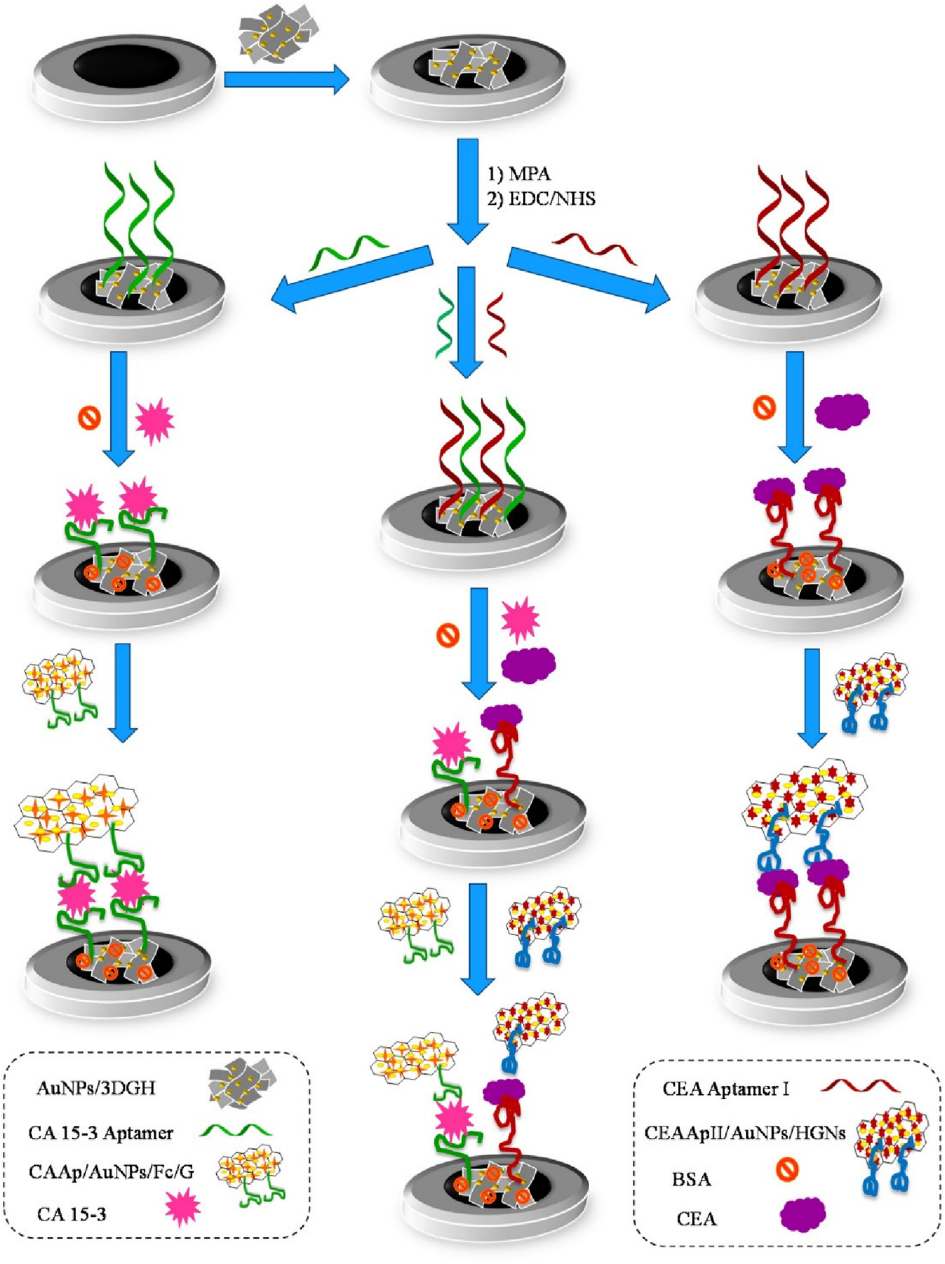
Schematic diagram of the different steps involved in the preparation of the sandwich–type aptasensor for the individual and simultaneous detection of CEA and CA 15–3 biomarkers (40). Zhara Shekari et al., Dual assaying of breast cancer biomarkers by using a sandwich–type electrochemical aptasensor based on a gold nanoparticles-3D graphene hydrogel nanocomposite and redox probes labelled aptamers. Copyright 2021, with permission from Elsevier.

Shekari et al. fabricated a sandwich-type electrochemical as depicted in Figure 9 aptasensor for the simultaneous detection of CEA and CA 15-3 (40). Herein, gold nanoparticles/3D-graphene hydrogel nanocomposite was modified with a biomarker-specific aptamer. This aptamer-modified hydrogel was grafted onto a glassy-carbon electrode and DPV was used for the determination of both calibration plots and LODs through a current response of solution hemin and ferrocene. The reported calibration plots were linear and in the concentration ranges of 1.0 × 10^−2^–75.0 ng/ml for CEA and 1.0 × 10^−2^–150.0 U/ml for CA 15-3. LODs were reported as 3.9 pg/ml and 5.8 × 10^−3^ U/ml. The reproducibility of the sensors was evaluated and reported to be satisfactory at a difference of 6.0% and 5.6% for CEA and CA 15-3, respectively. Storage was also tested and reported to display a degradation of 9% and 10% for CEA and CA 15-3, respectively, over 3 days.

### 3.2 Triplex devices

Electrochemistry can oftentimes be combined with a variety of varying sensing techniques to both increase the sensitivity of the prospective platform and allow for modularity of control over the platform parameters. Work published in 2020 by Cotchim et al. demonstrates the prototype of a multiplex electrochemically mediated immunosensor to detect breast cancer serum antigens CEA, CA153, and CA125 with anti-CEA, anti-CA153, and anti-CA125, respectively (41). This platform was designed to run on a single antibody-modified indium oxide glass electrode and harnessed cyclic voltammetry (CV) and electrochemical impedance spectroscopy (EIS) to characterize successful antigen binding. The redox responder in this prototype leveraged the use of a methylene blue-modified chitosan cryogel which was attached to the electrode surface. The multiplex sensor demonstrated a range of detection at 0.10–100.00 pg/ml, 0.10–100.00 mU/ml, and 0.10–100.00 mU/ml for CEA, CA153, and CA125, respectively. The limits of detection were found to be 0.04 pg/ml, 0.04 mU/ml, and 0.04 mU/ml for CEA, CA153, and CA125, respectively. Importantly, the cost of one multiplex device was evaluated to be at $0.86 USD and displayed robust reusability at 10 uses per sensor.

Pimalai et al. (42) have developed a multiplex electrochemical sensor for the detection of serum micro-ribonucleic acid (miRNA) cancer markers (miRNA-155, miRNA-21, and miRNA-16). Using antibody-modified screen-printed carbon electrodes, target mRNA molecules were detected through their binding to a DNA-modified silver-gold nanoparticle capture probe. Once bound, this DNA-modified capture probe would then subsequently attach to antibody markers which had been deposited on the electrode's surface. Additionally, to increase the sensitivity of each sensor, the capture probes were modified with Pb^2+^, Cd^2+^, and Cu^2+^ for miRNA-21, miRNA-155, and miRNA-16, respectively. This modification to the probes resulted in the amplification of the electrochemical response—herein measured by differential pulse voltammetry. Low limits of detection at 9.79 × 10^−16^ M, 3.58 × 10^−15^ M, and 2.54 × 10^−16^ M were obtained for miRNA-155, miRNA-21, and miRNA-16, respectively. However, as is perhaps a challenge with many screen-printed electrode-based platforms, long-term storage of these devices can be a barrier to bringing these types of platforms to market. The authors report a storage stability of 14 days; a great longevity of screen-printed electrodes in the lab but one that significantly impedes the sensors' viability as a sold product without significant resource allocation towards dedicated sensor production times and personnel.

Kuntamung et al. were able to develop a label-free electrochemical breast cancer sensor that targeted MUC-1, CA 15-3, and HER2 (43). To do so, gold nanoparticles were heavily modified with the presence of antibody-conjugated polyethyleneimine and subsequently cast onto a SPCE. Antibodies were then immobilized on the electrode surface and measured via sweeping wave voltammetry from −1.1 V to 0.8 V in a 0.010 M PBS/5.0 mM [Fe(CN)_6_]^3−/4−^ solution. The estimated LODs in solution for MUC-1, CA15-3, and HER2 were found to be 0.53 ng/ml, 0.21 U/ml, and 0.50 ng/ml, respectively. It is yet again worth noting that due to the nature of the fabrication of SPCE sensors, especially after the establishment of an SPCE modification procedure, a variability of up to 6.5% can be seen between the electrodes used by the research team. With this in mind, it is important to check these results against use in complex serum to ensure high degrees of both specificity and selectivity. In biomarker-spiked human serum, MUC-1, CA153, and HER2 were detected with a variable range of 96.69%–104.95%, 96.68%–104.05%, and 101.12%–105.48%, respectively, indicating the sensor's viable use in serum samples. Finally, to explore the storage viability of the sensor, the electrodes were prepped after 4 weeks of storage and displayed a variance of 10%. This indicates the potential for a brief shelf life but further investigations into long-term storage should be further explored for potential application to industry.

**Figure 10:**
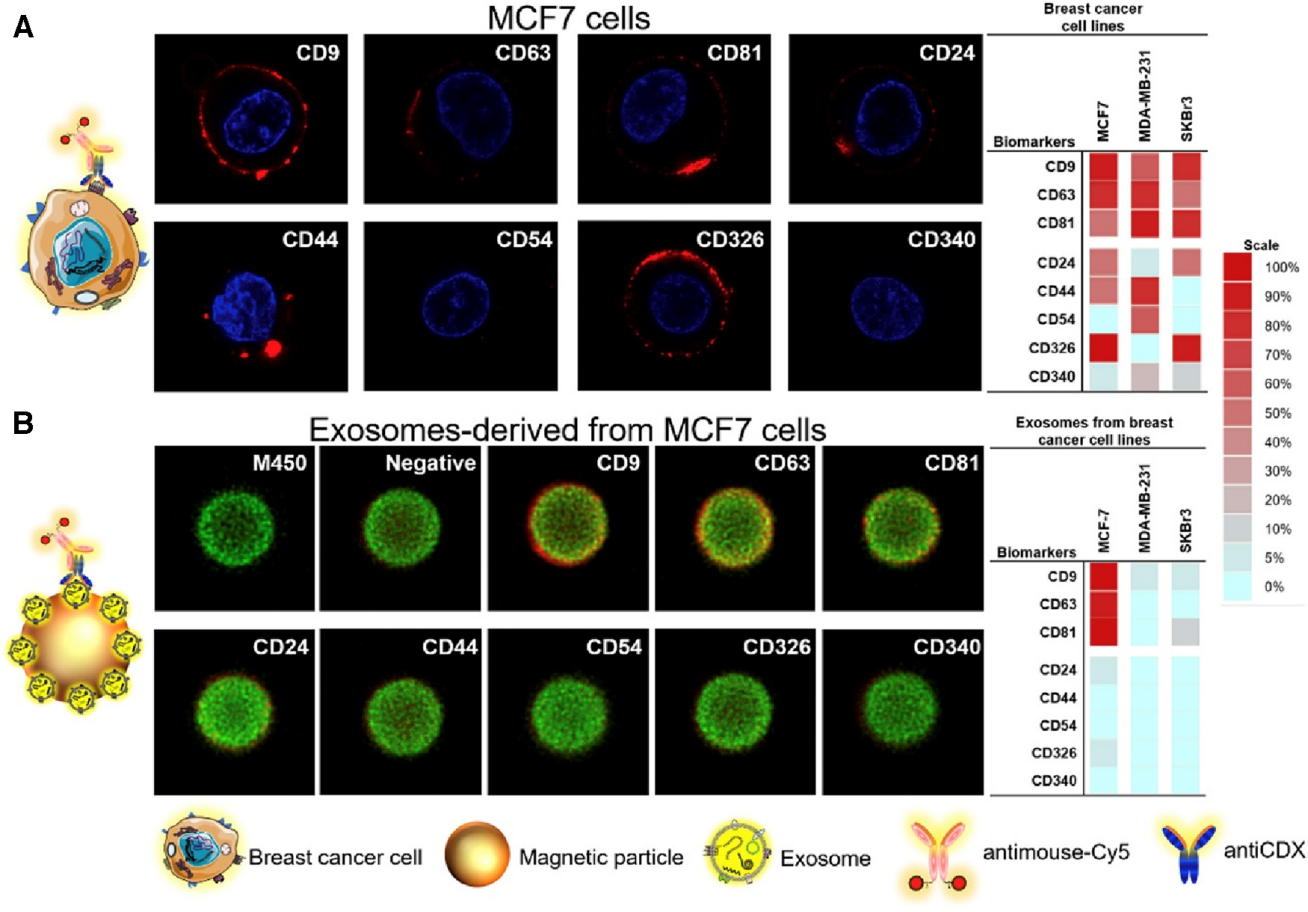
Confocal microscopy study for (**A**) MCF7 breast cancer cell lines and their corresponding exosomes covalently immobilized on MPs (exosomes-MPs), followed by indirect labelling with mouse antiCDX (being CDX either CD9, CD24, CD44, CD54, CD63, CD81, CD326 and CD340 biomarkers) and antimouse-Cy5. The concentration of exosomes was set at 4 × 10^9^ per assay. The scale indicates the percentage of positive entities (cells and exosomes-coated MPs in panels **A**,**B**, respectively. Silio Lima Moura et al. Electrochemical immunosensing of nanovesicles as biomarkers for breast cancer (44). Copyright 2020, with permission from Elsevier.

Moura et al. leveraged the use of magnetic particles (MPs) to detect the presence of breast cancer exosomes in both lab-mixed and donor serums (44). Herein, the authors explored the sensing of general exosome biomarkers (CD9, CD63, and CD81), and cancer-related biomarkers (CD24, CD44, CD54, CD326, and CD340) which could be identified on exosomal surfaces derived from the three distinct breast cancer cell lines (MCF-7, MDA-MB-231, and SK-BR-3). To do this, immunomagnetic separation of the exosomes was first completed via the use of antiCDX MPs and each was then labelled with antiCD63-HRP antibodies. Electrochemical measures were then used to determine the LOD of 65 exosomes/μl solution via amperometric measurement of the MPs at the electrode surface. In human serum, cancer biomarkers antiCD24 and antiCD340 displayed a detectable signal to exosomes spiked in a non-dilute exosome-depleted human serum with a LOD of 1.94 × 10^5^ exosomes/μl and 1.02 × 10^6^ exosomes/μl, respectively. In the serum of breast cancer patients, the researchers were able to display discrimination in the concentration of CD24 (1.8-fold) and CD340 (1.6-fold) between healthy donors and breast cancer individuals. By exploring the ratios of biomarker detection between breast cancer positive and negative serums, the researchers were further able to validate the potential use for electrochemical sensing on the blood serum of breast cancer patients. Unfortunately, there are no standardized levels of normal to produce exosomes in human breast tissue, creating a distinct challenge in applying exosome-based technologies to the forefront of cancer biosensing. Images of the breast cancer cell lines and their corresponding exosomes covalently immobilized on MPs were analyzed using confocal microscopy as shown in Figure 10.

The work completed by Zhang et al. targets the release of HER2-positive breast cancer cell (SK-BR-3) exosomes via the use of a multiplex CD63, HER2, and EpCAM aptasensor (45). The authors looked to deposit aptamers on the electrode surface for specific binding to the exosomal cancer targets. Next, differential pulse voltammetry was used in a 10 mM PBS solution to target electrochemical reactions formed by the activation of a methylene tag on HER2 aptamer and changes to the recorded reduction of ferrocene in solution due to the conformation change of EpCAM aptamer on the electrode surface. The lower and upper limit detection for SK-BR-3 exosomes was 3.4 × 10^3^–3.4 × 10^8^ particles/ml. Specificity was also explored by the authors for HER2-positive vs. HER2-negative cancers. It was found that HER2 protein was present in higher concentrations of SK-BR-3 cell exosomes when compared to MDA-MB-231(a cell line for mammary adenocarcinoma-1), MCF-7 (Mammary adenocarcinoma-1), and a control cell line. Additionally, EpCAM was shown to be overexpressed on breast cancer cells in comparison to non-cancerous breast cells. This distinction allows for further distinguishment between not only HER2-positive cancers but also between exosomes released from cancerous and non-cancerous tumour cells.

## 4 Conclusion

This perspective identifies several important areas of exploration for electrochemical diagnostics. With the prior developments summarized; we hope to answer the following questions:

1. Are the limits of detection (LODs) reported for multiplex electrochemical biosensors of clinical relevance and how do they compare to well-established methods like ELISA, FISH, or PCR?

Yes, the LODs do display competitive sensitivity and specificity against the established methods. When exploring the variety of electrochemical platforms prototyped from a broad spectrum of molecular receptor capture probes (including nucleic acid aptamer and antibodies), these new electroanalytical methods demonstrate LODs at a similar range—or significantly lower—to conventional methods. For example, when comparing the LOD of 10^−12^–10^−9^ g/ml for HER2 biomarker measured by ELISA (in plasma serum, cell supernatant, or cell lysate) (taking as examples the Human ErbB2/HER2 ELISA Kit or MagVigen™—Human HER2 On-Bead ELISA Kit), the best performing HER2-responsive electrochemical multiplex platforms show a LOD of 0.5 ng/ml (43). For glycoproteins, MUC-1, and CA 15-3, the LODs reported for the electrochemical multiplex sensor are significantly lower than testified using standard analytics. As such, multiplex biosensors report LODs of 0.53 ng/ml and 0.21 U/ml for MUC-1 and CA 15-3, respectively (43), or 5.8 × 10^−3^ U/ml for CA 15-3 alone (40). For reference, a MUC-1 protein concentration of 12.43 ng/ml is reported before chemotherapy and 11.69 ng/ml after chemotherapy (46). CA 15-3 is currently sensed at ≤30 U/ml in the current clinical blood tests (47).

The LOD for micro-ribonucleic acid (miRNA) biomarkers were reported to be 9.79 × 10^−16^ M, 3.58 × 10^−15^ M, and 2.54 × 10^−16^ M for miRNA-155, miRNA-21, and miRNA-16, respectively (42), and in the fM range for miRNA-21 (39). When compared to the standard qRT-PCR method used for the same miRNAs (ranging at ng/ml) (48), the sensitivities of the reported electrochemical multiplex assays were all notedly superior. The LOD of 10^3^–10^8^ particles/ml for exosomal BC markers quantified by the electrochemical assay by Zhang and co-workers (45) can also be regarded as a great success when compared to detection by the tracking and analysis of nanoparticles (∼ 10^7^ particles/ml) (49), flow cytometry, Western blotting and ELISA. However, the LOD declared for the e-multiplex sensors is comparable to other emerging breast cancer exosomal sensors measured by non-electrochemical methods such as colourimetry, fluorescence, surface-enhanced Raman scattering (15 particles/ul) (49), UV-Vis spectroscopy (LOD of 1.6 × 10^2^ particles/μl) (50), and other optical methods.

Finally, the ELISA assay demonstrates LODs of 78–5,000 pg/ml for RANKL and 16–1,000 pg/ml for TNF, and low threshold detectable concentrations of 20 pg/ml and 5 pg/ml for RANKL and TNF, respectively. The reported LOD values of 2.6 and 3.0 pg/ml for RANKL and TNF, respectively, are comparatively superior as demonstrated by the electrochemical dual immunoassay platform (29). Both EGFR and VEGF markers can be quantified at much lower concentrations (0.01 and 0.005 pg/ml for EGFR and VEGF, respectively) as compared to their respective assays performed by ELISA (EGRF at 0.31–20 ng/ml and VEGF at 31.3–2,000 pg/ml).

2. Can a single sensor electrode be used for the detection of multiple markers in a “one-blood-drop” fashion?

Not at the current state of development—particularly when nucleic acid aptamers are used as the sensor biorecognition elements. Many multiplex platforms use oligomers, such as aptamers, which are highly dynamic and versatile biorecognition elements that adopt a variety of 3-dimensional shapes and structures depending on the medium and matrix in which they are present. The sensitivity of aptamers to their environment and the variance in aptamer immobilization makes it challenging to achieve a uniform and steady state, sometimes leading to false positives caused by interferences. This is especially true after periods of electrode storage, and when interacting with high quantities of adjacent aptamer. Changes in the medium itself can additionally result in a more significant signal change than what might be caused by the target analyte. Even after more than twenty years of research on aptasensors, creating a single marketable electrochemical platform has proven to be a challenging endeavour. In addition to the complexity of aptamers, the target analyte biomolecules are also highly dynamic and exhibit an incredible range of functions and unique 3D conformations arising from their various primary, secondary, tertiary, and quaternary structures. These complex structures are often highly interdependent and are responsible for driving the molecule's function. Moreover, proteins exhibit complex inter- and intramolecular interactions that are difficult to study and quantify.

With this in mind, the most realistic solution for the electrochemical multiplex platform is to use electrode arrays in which each platform is engineered to detect a single analyte. Yet, it brings a question of cost and technical complexity. Fortunately, manufacturers of electrochemical accessories such as screen-printed electrode arrays (DropSense, ItalSense among many others) offer a good selection of array platforms that can be easily customized to adjust the number of markers being sensed. More importantly, these accessories are compatible with many electrochemical workstations allowing for simultaneous reading of electrical signals, all without sacrificing the size and adding to the cost of the electrochemical reader [MultiPalmSense4 or μStat-MultiX Multichannel (Bi)potentiostat/Galvanostat/Impedance Analyzer, MultiplEIS®]. Additionally, artificial intelligence-assisted machine learning algorithms are already allowing for simplified data processing (e.g., baseline correction, data standardization, and data compression) of electrochemical outputs. These tools will allow for the sensing of breast cancer biomarkers in real-time and could eventually help in delivering a POC single blood-drop analysis (51, 52). With this in mind, as soon as the receptor-analyte affinity and binding are optimized, these multiplex biosensors have the potential to be very successful POC devices for the early screening of BC.

3. What mechanisms of signal amplifications are most promising and what technological advancements are needed to utilize these devices for multiplex POC detection?

Any biorecognition element coupled with an amplification method, such as a dual rolling circle amplification (38), magnetic beads, metal nanoparticle, or enzymatic labels, has shown high potential for BC biomarker diagnoses and can allow for very low detection limits. However, these methods often require a multistep process for sample analysis; thus, they are more difficult to establish and are time-consuming. Though not as effective as molecular amplification, an appropriate and cost-effective alternative is magnetic enrichment which can be used for moderate levels of signal amplification.

4. Can nanotechnology advance the sensitive and selective diagnostics of multiple BC biomarkers?

*Nanomaterials-based electrochemical sensing*

It is readily apparent that implementing nanomaterials in the design of a sensing platform has several benefits. By implementing changes to the electrode features themselves, nanomaterials (metal and carbon nanoparticles and quantum dots) provide an increased active surface area and improved electrical conductivity, and thus an improved charge transfer ability of the platform. This is of particular note when high quantities of poor-conducting biological species are immobilized on the sensor platform. Furthermore, as demonstrated in the works surveyed in this review, metal nanoparticles are often used as redox responders and can be easily conjugated with biomolecules, such as gold self-assemblies via thiol motifs or through the coupling of the carbon functionalities with amines via EDC/NHS coupling. H. Nasrollahpour and colleagues (27) did remarkable work by discussing comprehensively how the integration of nanomaterials in various aspects of biosensor development can aggregate multiple benefits in the construction of electrochemical assay for monitoring breast cancer markers. The incorporation of various nanostructures in such an assay should always be for amplification of an electrochemical signal and when implemented correctly, forms the backbone for progression in the screening and diagnostic of BC.

*Micro-and nano-biosensors, microfluidics devices*

The development of micro- and nano-biosensors gives us a unique lens into the possible future of cancer screening, diagnostics, and in-real-time treatment management. For instance, in microfluidic devices can take small quantities of human serum and pump them through a small array of tubes (53). As the serum passes over the electrode surface, and the target analyte comes into contact with biorecognition molecules which had been deposited on the electrode surface, we can all but guarantee that binding will occur due to the repetitive flow of the serum over the biorecognition element. This allows for the development of highly specific and sensitive devices that could capture as little as a single cell or analyte. Additionally, these devices promise to be highly portable, needing only the microfluidics pump and tubing, the sensing platform, a laptop computer, and a potentiostat to run. Finally, these microfluidic systems could run in tandem, allowing for screening across any number of biomolecules at one time.

Without a doubt, non-specific binding of species present in biological fluids (serum, blood, plasma) is the main challenge that must be overcome to transfer electrochemical biosensors to the marketable product. The concentration of these interfering biomolecules is several orders of magnitude higher than that of target analytes. These non-target molecules simply adhere to the sensor surface without any specific receptor, thereby severely lowering the sensitivity and specificity of the assay. This problem must be solved before any further progress can be made towards clinical testing. One interesting concept that seeks for solution to the non-specific adsorption of proteins in a complex medium is referred to as nanoshearing (54, 55). Aided by an alternating (AC) electric field, shear forces are generated near the electrode surface. Via manipulation of AC field and thus the vector of these forces, the selectivity of the assay can be improved. Specifically, selective displacement of weakly (non-specifically) bound proteins from the electrode surface is possible by tuning the fluid shear forces. Using this approach, the detection of HER2 BC marker and other multiple protein targets spiked in human serum can be significantly improved (1,000-fold sensitivity enhancement) in comparison to other flow-based assays for protein biomarker detection.

5. Are there preferred receptors (antibody, nucleic acid or their combinations) and preferred biosensor designs (complementary methods, sandwich-type protocols, and antibody/aptamer concept, label-free protocol) that can aid in clearing the roadmap for early diagnosis of multiple BC markers?

In this review, we have surveyed different approaches, which include using diverse techniques, bioreceptors, bioassays, electrode types, and signal amplification strategies applied towards the robust sensing of BC. These methods range from simple, label-free tests to complex multistep procedures, but each provides attractive approaches that enable better biomarker recognition. However, it is not yet clear which of these approaches are reliable for sensitivity and selectivity since many of the models are not validated by others, nor are they tested clinically. More research needs to be carried out to cross-reference the fabrication and analytical response with minimum error for these multianalyte biosensors. Furthermore, there exists new innovative—but more expensive methods that have not yet been explored for multi-marker breast cancer detection. This includes the use of Slow Off-Rate Modified Aptamers (SOMAmers) as biorecognition elements, which could potentially improve the selectivity of the biorecognition element. Monomeric or zwitterionic peptides used as bio-recognition elements or electrode modifiers could effectively prevent unfavourable binding of non-specific proteins and cells. Several biosensing platforms which have been tested in human serum have been reported using the remarkable antifouling ability of peptides by Luo and co-workers with promising results (56–58). Even with these advancements, other technologies such as CRISPR-based electrochemical biosensing could lead to further advancement in BC and cancer recognition. The simultaneous detection of 37 RNAs with 95.2% specificity and 90.0% sensitivity from human serum of early-stage cell lung cancer patients has been shown by Sheng and colleagues (56). This approach could evolve as a next-generation diagnostic tool for screening serum BC biomarkers.

6. Why are we still without FDA-approved electrochemical multiplex devices for BC screening?

There is only one FDA-approved, PCR-based multiplex testing kit, manufactured by Myriad Genetic Laboratories (BRACAnalysis CDx), which specifically targets BRCA1 and BRCA2 markers for breast, ovarian, and pancreatic cancers (https://myriad.com/genetic-tests/bracanalysiscdx-germline-test/). If the above-discussed issues for the multiplex e-sensors are solved, these devices have the potential to meet the POC criteria, resulting in efficient, rapid, and smart multiplex serological tests for BC markers only.

In addition, to make strides towards commercialization and significant research advancement we must see a change in the reporting culture in sensing manuscripts. Currently, there is an overarching failure by researchers to utilize and report fundamental statistical concepts in every step of biosensor design and fabrication including the omission of error calculations; a lack of significant tests that demonstrate sensor validity and viability such as baseline charge-signal drift and the effects of long-term device storage on signal transduction; and a lack of a robust statistical design of experiments. As an example, the effectiveness of aptamer-based biosensors can significantly depend on experimental conditions such as salt concentration, buffer, and pH value, each of which has been found to affect the selectivity, affinity, and 3D structure of aptamers. Despite this, high-resolution structures of aptamer complexes have been determined under different experimental conditions with no apparent relationship between them. The DoE approach considers all factors simultaneously and provides an empirical correlation between these factors on the chosen response, unlike the conventional method of changing one factor at a time. The evaluation of experimental variables, stability of the assay, selectivity towards all possible interfering species, and finally testing in complex matrix (blood, serum, liquid biopsy) verified by both a newly developed electrochemical assay and tested against the gold standard of analytics (ELISA, PCR, FISH, etc.) are direly needed if these technologies are to make an impact in real-world settings.
